# Immunization with different formulations of *Mycobacterium tuberculosis* antigen 85A induces immune responses with different specificity and protective efficacy^☆^

**DOI:** 10.1016/j.vaccine.2013.07.040

**Published:** 2013-09-23

**Authors:** Elma Tchilian, Diksha Ahuja, Ariann Hey, Shisong Jiang, Peter Beverley

**Affiliations:** a University of Oxford, The Peter Medawar Building for Pathogen Research, South Parks Road, Oxford OX1 3SY, UK; b University of Oxford, Department of Oncology, Old Road Campus Research Building, Rooseveldt Drive, Oxford OX3 7DQ, UK

**Keywords:** Adenovirus, Antigen 85A, Epitopes, Subunit vaccine, Tuberculosis

## Abstract

To test the relative efficacy of CD4 and CD8T cells in mediating protective immunity to *Mycobacterium tuberculosis* (*Mtb*), we compared three immunization regimes designed to induce preferentially each subset. BALB/c mice were immunized intranasally (i.n.) or parenterally with antigen 85A either in a recombinant Adenoviral vector (Ad85A), as recombinant protein (r85A) or as a set of overlapping 15mer peptides (p85A). For the first time we show that i.n. immunization with overlapping 85A synthetic peptides as well as Ad85A or r85A can provide protection against *Mtb* challenge. For all forms of the antigen, i.n. induces greater protection against *Mtb* challenge than parenteral immunization. Ad85A induces a predominantly CD8T cell response against the 85A_70–78_ epitope, r85A a CD4 response to 85A_99–118_ and p85A a balanced CD4/CD8 response to the CD4 85A_99–118_ and CD8 85A_145–152_ epitopes. Immune responses to CD4 85A_99–118_ and CD8 85A_70–78_ but not CD8 85A_145–152_ are protective. Although Ad85A induces a strong response to the protective CD8 85A_70–78_ epitope, we could not induce any response to this epitope by peptide immunization. These results show that although peptide immunization can induce protective immunity to *Mtb* challenge, it can also induce a response to a non-protective epitope in antigen 85A, indicating that the specificity of an immune response may be more important for protection against *Mtb* than its magnitude. These findings have important implications for the application of such vaccines in humans.

## Highlights

• Immunization intranasally with *Mtb* antigen 85A is more protective than parenterally.
• Three 85A vaccines platforms induce responses with differing epitope specificity.
• Responses to the CD8 85A_70–78_ but not the CD8 85A_145–152_ epitope are protective.

## 1 Introduction

Tuberculosis (TB) remains an important disease worldwide and new vaccines to replace or boost the protection afforded by the existing vaccine, Bacille Calmette Guérin (BCG), are much needed, especially in view of the development and spread of multidrug resistant strains of *Mtb*. Members of the mycolyl transferase family of *Mtb* proteins, also called the antigen 85 complex, are amongst the most prominent tuberculosis vaccine candidates [1] because immune responses to these antigens can be detected in the majority of humans exposed to *Mtb* [2]. Numerous studies have shown that immunization with antigen 85 complex proteins, using a variety of vaccine platforms, can provide protection against *Mtb* challenge in animal models [1,3–6].

We have used antigen 85A as a model vaccine antigen to explore in mice how to induce protection against *Mtb* challenge and define the features and mode of action of protective immune responses. These experiments have shown that intranasal (i.n.) immunization is a highly effective route of administration [6,7] inducing an activated population of lung resident T cells that inhibit mycobacterial growth very early after *Mtb* challenge, in contrast to the later effect of parenteral immunization [6,8–11]. Furthermore, under some circumstances mucosal and parenteral immunization can have additive protective effects [6,10].

Although recent studies are beginning to define the characteristics of protective pulmonary mucosal immune responses [12] and need for pathogen associated molecular pattern signals for appropriate innate immune system activation [13,14], many questions remain to be resolved, particularly the relative importance of CD4 and CD8T cells in protection against early mycobacterial growth established by pulmonary immunization. In this study we compare the protective efficacy of immunization protocols designed to induce CD4 or CD8 biased or balanced CD4/8T cell responses to the single *Mtb* antigen, 85A. We show that the CD4 response to 85A is protective but that CD8T cell responses may be protective or non-protective, depending on their epitope specificity.

## 2 Materials and methods

### 2.1 Mice and immunization

**Fig. 1:**
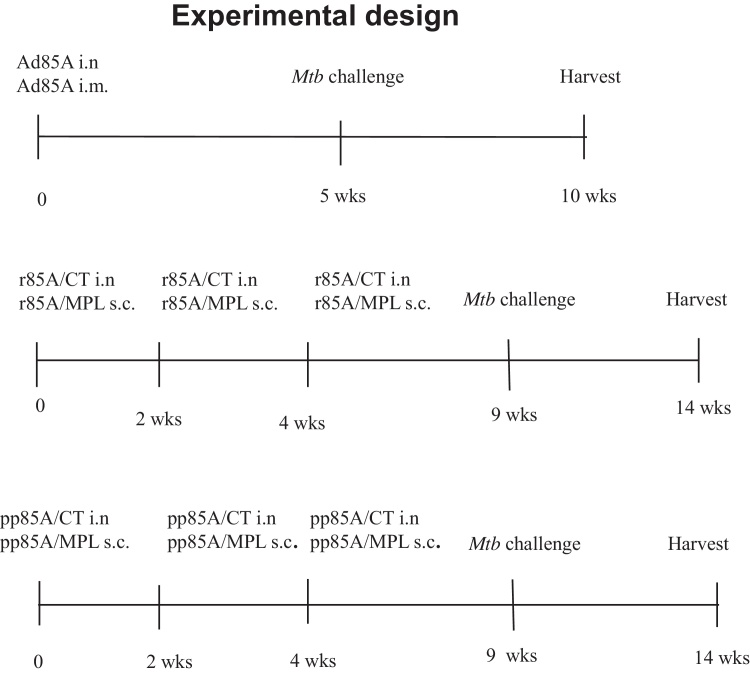
Experimental design. Horizontal lines indicate the time scale of the experiments and the vertical bars show immunizations, *Mtb* challenge and harvest for CFU assay.

All experiments were performed with 6–8 week old female BALB/c mice (Harlan Orlac, Blackthorn, UK), were approved by the animal use ethical committee of Oxford University and complied with UK Home Office guidelines. Mice were immunized as shown in Fig. 1, with either recombinant human serotype 5 adenovirus expressing 85A (Ad85A) [9], recombinant 85A protein (r85A) or with 15mer peptides overlapping by 10 amino acids covering the entire protein sequence of antigen 85A (p85A) (Suppl. Table 1).

Table S1 Sequences of antigen 85A overlapping peptides. The table shows the sequences of 15mers overlapping by 10 amino acids, covering the sequence of *Mtb* antigen 85A.

#### 2.1.1 Ad85A

Mice were immunized once with Ad85A. For i.n. immunization, mice were anaesthetized with isoflurane/oxygen and 2 × 10^9^ virus particles (vp) of Ad85A in 40 μl of PBS was administered with a pipette divided between both nostrils. For intramuscular (i.m.) immunization 2 × 10^9^ vp of Ad85A was administered in the hind legs in a total volume of 50 μl.

#### 2.1.2 r85A

Mice were immunized three times at 2 week intervals with 2 μg r85A protein (a kind gift from KLMC Franken and THM Ottenhoff) prepared as described previously [10,15]. For i.n. immunization mice were anaesthetized with isoflurane/oxygen and 2 μg r85A was mixed with 2 μg of cholera toxin (CT) (Sigma, UK) and pipetted into the nostrils in a total volume of 40 μl. Mice were also immunized subcutaneously (s.c.) with 2 μg of r85A in 200 μl of monophosphoryl lipid A (MPL) (Sigma, UK).

#### 2.1.3 p85A

Mice were immunized three times at 2 week intervals with 500 ng of each of the sixty-six 15mer peptides overlapping by 10 amino acids covering the entire sequence of antigen 85A (p85A) as described for r85A. For immunization against CD4 85A_99–118_ 1 μg of each of the four peptides covering the region containing the 99–118 epitope, ETFLTSELPGWLQAN, SELPGWLQANRHVKP, WLQANRHVKPTGSAV and RHVKPTGSAVVGLSM (peptides 29, 30, 31 and 32 in Suppl. Table 1), were administered with adjuvant i.n. or s.c. as above.

For immunization against CD8 85A_70–78_ 1 μg of each of the three peptides covering the region containing the 70–78 epitope QSGLSVVMPVGGQSS, VVMPVGGQSSFYSDW, GGQSSFYSDWYQPAC (peptides 22, 23 and 24 in Suppl. Table 1) were administered with adjuvant i.n. or i.m. as above.

For immunization against CD8 85A_145–152._ 1 μg each of the two peptides covering the region containing the 145–152 epitope, YHPQQFVYAGAMSGL and FVYAGAMSGLLDPSQ peptides (peptides 37 and 38 in Suppl. Table 1) were administered with adjuvant i.n. or s.c. as above.

Mice were also immunized either with all peptides except the CD4 85A_99–118_ region (all peptides without peptides 29, 30, 31 and 32), all peptides except the CD8 85A_70–78_ region (lacking peptides 22, 23 and 24) or without the CD8 85A_145–152_ region (lacking peptides 37 and 38) or in combinations as in the text.

### 2.2 Isolation of lymphocytes from lungs and spleen

Lungs were perfused with PBS, cut into pieces and digested with 0.7 mg/ml collagenase type I (Sigma) and 30 μg/ml DNase I (Sigma) for 45 min at 37 °C. Digested fragments were crushed through a cell strainer using a syringe plunger, washed, layered over Lympholyte (Cederlane, Ontario, Canada) and centrifuged at 1000 × *g* for 25 min. Interface cells were collected and washed. Spleens were passed through a cell strainer using a syringe plunger, red blood cells were removed with lysis buffer (Qiagen, Crawley, UK) and the cells were washed.

### 2.3 Flow cytometry

Cells were cultured in Hepes buffered RPMI, 10% heat-inactivated FCS, L-glutamine, penicillin and streptomycin for 6 h. Cells from Ad85A immunized animals were stimulated for 6 h with either sixty-six 15mer peptides overlapping by 10 amino acids covering the 85A protein sequence or with pool of peptides containing about 10 consecutive peptides or with 3 peptides (Peptide Protein Research Ltd., Fareham, UK) encoding the dominant CD4 (Ag85A_99–118aa_ TFLTSELPGWLQANRHVKPT) and CD8 (Ag85A_70–78aa_ MPVGGQSSF and Ag85A_145–152aa_ YAGAMSGL) peptide epitopes [9] (Peptide Protein Research Ltd). Each peptide was at 2 μg/ml. After 2 h at 37 °C, Golgi Plug (BD Biosciences, Oxford, UK) was added according to the manufacturer's instructions.

Cells were washed and incubated with CD16/CD32 monoclonal antibody to block Fc binding. Subsequently the cells were stained for CD4 (RM4-5), CD8 (53-6.7) (BD Bioscience, Oxford, UK), IFNγ (XMG1.2) (eBioscience, Hatfield, UK) using the BD Cytofix/Cytoperm kit according to the manufacturer's instructions. Cells were run on a LSRII (BD Biosciences) and analyzed using FlowJo software (Tree Star Inc., Ashland, Oregon, USA). For simplicity IFNγ intracellular staining was used to detect the majority of antigen specific CD4 and CD8T cells, since the experiments were concerned with the specificity of the response to 85A and not its quality.

### 2.4 Infection with *Mtb* and determination of mycobacterial load

Five to 7 mice were anesthetized with isoflurane/oxygen and infected i.n. with *Mtb* (Erdman strain) in 40 μl PBS. Lung CFU were enumerated 24 h after challenge to determine the number of organisms deposited (∼200 CFU). Mice were sacrificed at indicated times, the lungs and spleen homogenized and mycobacterial load determined by plating 10-fold serial dilutions of tissue homogenates on Middlebrook 7H11 agar plates (E&O Laboratories Ltd., Bonnybridge, UK). Colonies were counted after 3–4 weeks of incubation at 37 °C in 5% CO_2_.

### 2.5 Statistical analysis

Data were analyzed using one-way ANOVA followed by Tukey's multiple comparison test.

## 3 Results

### 3.1 Immunization with 85A in three different vaccine platforms is more protective intranasally than parenterally

We first examined protection against *Mtb* challenge afforded by antigen 85A administered either as Ad85A, r85A or p85A (Fig. 1).

**Fig. 2:**
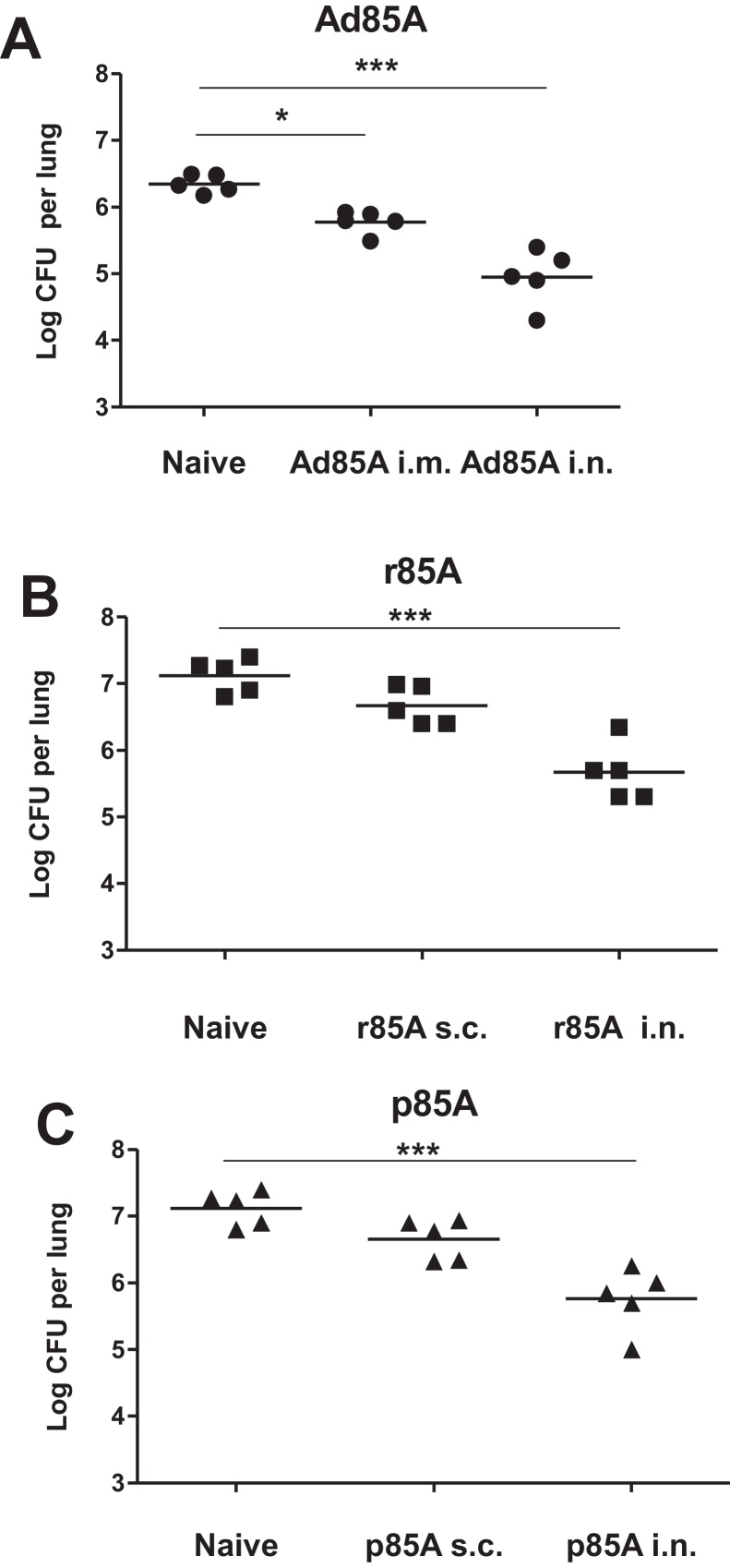
Protective efficacy of antigen 85A administered i.n. or parenterally. Unimmunized, i.n. or parenterally immunized BALB/c mice were challenged 5 weeks after the last immunization with *Mtb* i.n. and after further 5 weeks sacrificed for enumeration of lung and spleen (not shown) *Mtb* CFU. (A) Ad85A was administered i.n. or i.m. without adjuvant. (B) r85A was administered i.n. with cholera toxin or s.c. with MPL. (C) p85A were administered i.n. with cholera toxin or s.c. with MPL. Data from one of three experiments with 5 mice/group are shown. ***p < 0.001, *p < 0.05 compared to naïve animals, one-way ANOVA with Tukey's post test.

Mice immunized with Ad85A i.n. or i.m., were challenged after 5 weeks and *Mtb* lung CFU assessed 4 weeks later. The lung mycobacterial load is reduced by ∼0.6 log_10_ in Ad85A i.m. immunized compared to naïve animals, while Ad85A i.n. mice show an ∼1.3 log_10_ reduction (Fig. 2A). This is consistent with previous observations showing superior protection of i.n. administered Ad85A compared to parenteral routes [6,7].

To determine the protection afforded by r85A, BALB/c mice were immunized with r85A either i.n. or s.c. as shown in Fig. 1 and challenged five weeks after the last immunization. Intranasal immunization with r85A decreases the mycobacterial load by ∼1.5 log_10_ compared to naive mice, while parenteral immunization has an insignificant effect (Fig. 2B). Similar results are obtained when the mice are immunized with p85A. The i.n. immunized animals show a significantly reduced *Mtb* load while s.c. immunization has an insignificant effect (Fig. 2C). We also immunized mice 3 times with CT i.n. or MPL s.c. and challenged them with *Mtb*. The mycobacterial burden in these controls does not differ from that found in naive animals (data not shown).

Taken together these data show that the i.n. route of administration of Ad85A, rec85A or p85A, is more effective against *Mtb* challenge than parenteral immunization in BALB/c mice when CT and MPL are used as the mucosal and parenteral adjuvants.

### 3.2 Specificity of the immune responses to 85A administered in different vaccine platforms

The same antigen was administered in all immunization platforms and the outcome in terms of protection was similar in all i.n. immunized mice. However, since Ad85A induces a predominantly CD8T cell [6,9,11] while recombinant proteins in adjuvant induce CD4T cell responses [5,16,17] and 15mer peptides might induce both CD4 and 8 responses [18], we investigated the specificity of the immune responses to Ad85A, r85A and p85A.

Peptide pools covering the whole sequence of antigen 85A protein were used to stimulate lung and spleen cells from immunized mice for 6 h *in vitro* and intracellular staining for IFNγ was performed to detect the majority of antigen specific CD4 and CD8T cells (data not shown). After identifying the response to a pool, the exact sequence of the peptide was narrowed down using individual 15mer peptides overlapping by 10 amino acids. We identified three major immunodominant epitopes: CD4 85A_99–118_, CD8 85A_70–78_ and CD8 85A_145–152_ as previously shown for antigen 85A in H-2^d^ mice [19]. However the different vehicles induce very different responses.

**Fig. 3:**
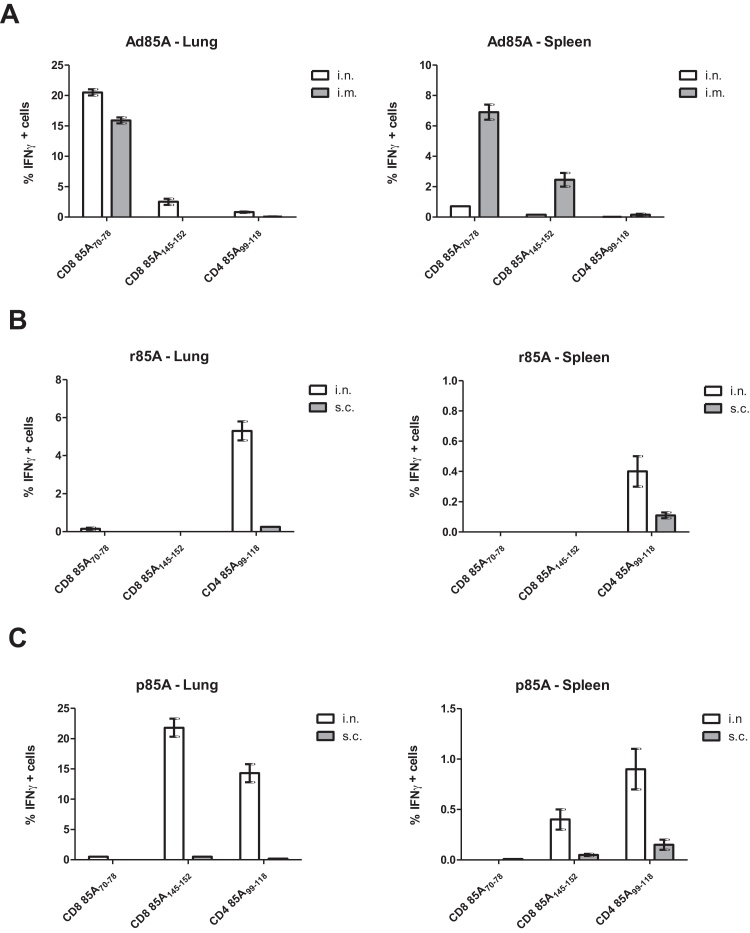
Specificity of immune responses to antigen 85A. Parenterally or i.n. immunized BALB/c mice were sacrificed 4 weeks after the last immunization and the proportion of lung and spleen CD4 and CD8 lymphocytes producing IFNγ determined by intracellular cytokine staining after 6 h stimulation with the 85A_99–118_, 85A_70–78_ and 85A_145–152_ peptides. (A) Ad85A was administered i.n. or i.m. without adjuvant. (B) r85A was administered i.n. with cholera toxin or s.c. with MPL. (C) p85A were administered i.n. with cholera toxin or s.c. with MPL. Cells from 3 to 4 mice were assayed separately in at least 5 experiments. Error bars show SD.

After Ad85A immunization most of the CD8 responding cells in the lungs and spleen react to the dominant 85A_70–78_ epitope and a smaller proportion to the 85A_145–152_ epitope. As previously shown there is also a small but detectable CD4 response to the 85A_99–118_ epitope. While the administration of Ad85A i.n. induces marginally stronger lung responses, the i.m. route induces stronger splenic CD8 85A_145–152_ and CD4 responses (Fig. 3A). In all figures, for simplicity we present only IFNγ data for CD4 and CD8 cells. When we used staining for TNF slightly fewer antigens specific were detected but the relative magnitude of responses to different epitopes was unchanged, while IL-2 stained few CD8T cells but could be used to detect CD4 antigen specific cells, giving similar results to IFNγ for this subset (data not shown).

Administration of r85A i.n. induces an ∼5% lung CD4 response against 85A_99–118_ but hardly detectable CD8 responses and this route induces larger responses not only in the lung but also in the spleen (0.4%) compared to s.c. immunization (0.1%) (Fig. 3B). Minimal and inconsistent CD4 responses are also detected against the 85A_141–156_ (FVYAGAMSGLLDPSQ peptide 38), 85A_178–193_ (DMWGPKEDPAWQRND peptide 45) or 85A_205–214_ (GKLIANNTRVWVYCG peptide 49) epitopes (data not shown), in accordance with previous studies in BALB/c mice (Suppl. Table 1) [19].

Animals immunized i.n. with p85A generate strong lung responses against the CD4 85A_99–118_ (∼23%) and CD8 85A_145–152_ (∼23%). Surprisingly no response to the CD8 85A_70–78_ epitope is detected. As with the other two regimes, i.n. immunization induces a stronger lung response compared to the parenteral route. Furthermore p85A i.n. induces a larger splenic response (∼1% CD4 and 0.3% CD8) compared to parenteral administration of p85A (∼0.1% CD4 and 0.05% CD8) (Fig. 3C).

### 3.3 Protective efficacy of CD4 85A_99–118_ and epitopes CD8 85A_145–152_ against *Mtb* challenge

**Fig. 4:**
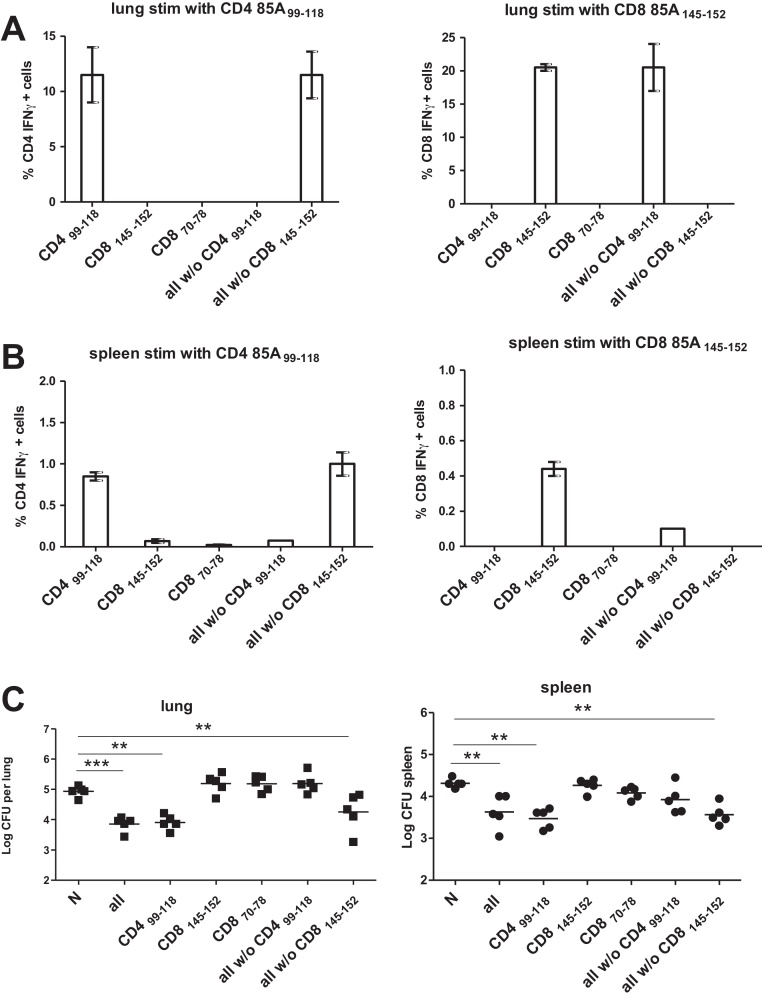
Immune responses and protection in peptide immunized mice. Mice were immunized i.n. with pools of 2–3 15-mer peptides covering the 85A_99–118_, 85A_70–78_ and 85A_145–152_ or peptide pools covering the whole 85A sequence from which the 85A_99–118_ or 85A_145–152_ sequences had been removed, as indicated on the x axis. 4 weeks after the last immunization the proportion of lung (A) and spleen (B) CD4 and CD8 lymphocytes producing IFNγ was determined by intracellular cytokine staining after 6 h stimulation with the 85A_99–118_ and 85A_145–152_ peptides. Cells from 3_–_4 mice were assayed separately. Error bars show SD. Similarly immunized mice were challenged with *Mtb* five weeks after the last immunization and lung and spleen CFU enumerated (C). Data from one of two experiments with 5 mice/group are shown. ***p < 0.001, **p < 0.01 compared to naïve animals, one-way ANOVA with Tukey's post test.

Since p85A i.n. induces both strong CD4 85A_99–118_ and CD8 85A_145–152_ specific responses we wished to determine the contribution of each of these epitope to protection against *Mtb*. We therefore immunized mice with peptides covering the CD4 85A_99–118_ or CD8 85A_145–152_ regions. In both cases the expected immune responses, to CD4 85A_99–118_ or CD8 85A_145–152_ are induced (Fig. 4A and B). The mice responding only to the CD4 85A_99–118_ epitope are protected as well as those immunized with all peptides, but strikingly, animals responding only to CD8 85A_145–152_ are not (Fig. 4C). Additional immunizations with all peptides minus the CD4 85A_99–118_ or CD8 85A_145–152_ peptides confirm that only an immune response to the CD4 85A_99–118_ epitope confers protection (Fig. 4C). Mice immunized with peptide pools lacking specific epitopes show antigen specific response identical to those immunized only with the CD4 85A_99–118_ or CD8 85A_145–152_ peptides (Fig. 4A and B).

No response to the CD8 85A_70–78_ epitope is detected in mice immunized with all peptides (p85A Fig. 3) or with peptides covering only this epitope (either as overlapping 15mers or a 9mer). Nor do mice immunized with all peptides without CD8_145–152_ or all without CD8_145–152_ and CD4 85A_99–118_, make a CD8 85A_70–78_ response. Finally, although we could not induce detectable CD8 85A_70–78_ responses, we still challenged these animals with *Mtb* and they show no protection (data not shown).

### 3.4 Activation status of 85A specific T cells

**Fig. 5:**
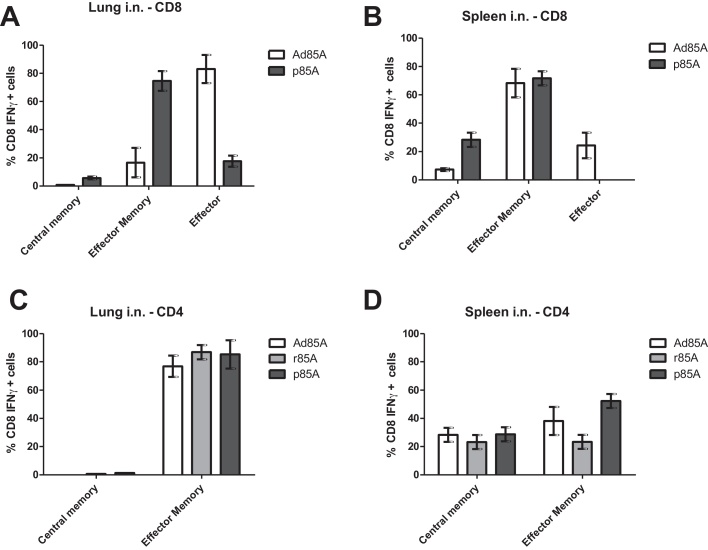
Activation status of antigen specific CD4 and CD8T cells. Mice were sacrificed 4 weeks after the last i.n. immunization with Ad85A, r85A or p85A and after 6 h stimulation of lung and spleen with 15mer peptides covering the 85A_99–118_, 85A_70–78_ and 85A_145–152_ sequences, the activation status of CD8T cells producing IFNγ was determined by staining with CD62L and CD127 (A and B) and that of CD4T cells producing IL-2 by staining with CD62L and CD44 (C and D). The proportions of central (CD62L+ CD127+) or effector (CD62L− CD127+) memory or effector (CD62L− CD127−) antigen specific CD8T cells after Ad85A and p85A immunization are shown for lung (A) and spleen (B). Proportions of lung and spleen central (CD62L+ CD44lo) and effector (CD62L− CD44hi) memory antigen specific CD4T cells after different i.n. immunizations are shown in (C) and (D), respectively. Cells from 4 mice were assayed separately. Error bars show SD. Representative data from one of three experiments are shown.

Because protective local lung immunity is associated with the presence of activated T cells in the lungs [8,9,11], we examined the activation status of antigen specific lung and spleen lymphocytes in immunized mice. Ad85A i.n. induces a higher proportion of effector (CD62L− CD127−) and effector memory (CD62L− CD127+) 85A specific CD8 cells in the lung and spleen compared to p85A i.n. (Fig. 5A and B). Most of the antigen specific lung CD4 cells after i.n. immunization are effector memory phenotype (CD62L− CD44hi), while in the spleen both effector and central memory (CD62L+ CD44lo) cells are seen, irrespective whether Ad85A, r85A or p85A are used for immunization (Fig. 5C and D).

In all cases i.n. administration induces a more activated phenotype of lung and splenic 85A specific cells, compared to the respective parenteral regime (data not shown).

Taken together the results demonstrate that the three delivery platforms and routes of administration induce antigen specific cells with different specificity and activation status.

## 4 Discussion

We assessed the immunogenicity and protective efficacy of *Mtb* antigen 85A administered as Ad85A, adjuvanted r85A or adjuvanted p85A and show that all formulations delivered i.n. reduce the lung mycobacterial load, despite the induction of different T cell subsets with differing epitope specificity. For the first time we show that i.n. administration of overlapping synthetic peptides covering the whole sequence of antigen 85A confers protection against *Mtb* challenge. The data do not establish however, that r85A or p85A are poor parenteral vaccines since it is possible that an improved adjuvant, such as those used by others with recombinant proteins, might improve the protection attained by parenteral immunization [5,20].

In accordance with previous observations in BALB/C mice [19,21], we find that antigen 85A induces responses to three immunodominant epitopes: I-E^d^ CD4_99–118_, L^d^ CD8_70–78_ and K^d^ CD8_145–152_. Ad85A induces a predominantly CD8T cell response mainly against the CD8 85A_70–78_ epitope, with minimal but detectable CD8 85A_145–152_ and CD4 85A_99–118_ responses. As expected, adjuvanted r85A induces a CD4 response against the dominant I-E^d^ CD4_99–118_ epitope, while p85A immunization induces both CD4 85A_99–118_ and CD8 85A_145–152_ responses. For all regimes the antigen specific cells in the lung are more activated compared to the spleen, with Ad85A inducing the most highly activated CD8 cells. These data demonstrate that irrespective of the T cell subset, the presence of highly activated effector/effector memory antigen specific cells at the site of infection may limit *Mtb* infection.

Although p85A induces responses to both CD4 85A_99–118_ and CD8 85A_145–152_, immunization with the separate peptides allowed us to determine their contribution to *Mtb* protection and show that only the CD4 response is protective. This may be because while CD4 85A_99–118_ and CD8 85A_70–78_ are expressed and can be recognized in *Mtb* infected cells, CD8 85A_145–152_ is not expressed. Because responses to all three epitopes are induced by Ad85A (Fig. 3), all three must be expressed in Ad85A infected cells and both the CD8 epitopes can also be detected in transfected P815 cells [22], implying that processing of antigen 85A may be different in *Mtb* infected macrophages. Such changes in epitope expression in different cells when different vaccine vectors are used, are not unknown; an extreme example is the recent finding of MHC class II restricted epitopes recognized by CD8T cells in Macaques immunized with a recombinant rhCMV vector [23].

Another example of changed epitope recognition in a mycobacterial antigen after different immunizations has been described previously in C57Bl/6 mice [16]. An adenoviral construct expressing an Ag85B/ESAT6 fusion gene induced a strong CD8 immune response to the ESAT6_15–29_ epitope but no protection. In contrast, immunization with IC31-adjuvanted recombinant protein induced primarily a CD4 response targeted to the Ag85B_241–255_ epitope and efficient protection against *Mtb* infection. This result too, was ascribed to lack of expression of the ESAT6_15–29_ epitope during *Mtb* infection.

An alternative explanation might be that because CD8 85A_145–152_ specific cells induced by peptide immunization are less activated than those induced by Ad85A (Fig. 5C and D), they are unable to mediate protection. Furthermore, CD8 85A_145–152_ on its own without CD4 help might induce defective CD8 memory cells [24,25] although clearly 85A_145–152_ specific cells efficiently produce IFNγ (and TNF, not shown) in response to antigen (Fig. 3).

A further puzzle in these experiments is why we cannot induce an immune response to the CD8 85A_70–78_ epitope by peptide immunization, while this is the dominant epitope detected following Ad85A administration and after DNA vaccination [22]. Since CD8 85A_70–78_ is an L^d^ and CD8 85A_145–152_ is a K^d^ epitope [19] it seems unlikely that competition of peptides for MHC class I binding, is the explanation. Furthermore responses to both epitopes are seen in Ad85A immunized (Fig. 3) or DNA immunized mice [22]. Nevertheless, even when CD8 85A_145–152_ is removed from the peptide immunization mix there is still no response to 85A_70–78_. It also seems unlikely that the lack of response is due to lack of CD4 help, as we tried immunizing with a mixture of the CD8 85A_70–78_ and CD4 85A_99–118_ epitopes.

Interestingly a longer peptide covering the sequence 141–160, in which the CD8 85A_145–152_. epitope is located, can stimulate cytokine secretion from both CD8 and CD4 cells as shown by others [19] and sporadically in our experiments. The presence of a cryptic CD4 epitope might contribute to the powerful response to the CD8 85A_145–152_ epitope during peptide immunization. As this region of 85A is recognized by T cells from 90% of *Mtb* infected humans [2], it may also be among the conserved epitopes, responses to which may benefit *Mtb* rather than the host [26].

In the light of the recent failure of the parenteral booster vaccine MVA85A to improve protection against tuberculosis over BCG alone, new vaccine strategies are urgently needed [27]. Immunization *via* the respiratory tract is promising. BCG administered by this route is protective in mice, guinea pigs, cows and primates [10,28–30] and subunit vaccines are protective in mice [4,6] and immunogenic in primates [31]. Peptides with an appropriate adjuvant offer a possible alternative formulation for respiratory administration, with the advantage that immunogenic epitopes can be selected and non-protective ones omitted from the vaccine. However, as shown here, some protective epitopes are not immunogenic when given as peptides. Much further work will be required to identify effective adjuvants that might be used in humans and to investigate the duration of protection after peptide immunization.

## 5 Conclusions

The three vaccines Ad85A, r85A and p85A are all protective when given i.n. but induce different T cell responses. Reponses to the CD4 85A_99–118_ and CD8 85A_70–78_, but not the CD8 85A_145–152_ epitope are protective. Peptide immunization did not induce an immune response to the protective CD8 85A_70–78_ epitope. These data have important implications for subunit vaccine design and show that the exact specificity of the T cell response to a vaccine antigen may be more important than its magnitude. It remains to be seen whether under other circumstances non-protective CD4 responses may be induced.
