# Supplementary material for: Immunization with different formulations of Mycobacterium tuberculosis antigen 85A induces immune responses with different specificity and protective efficacy
Source: Vaccine. 2013 Sep 23;31(41):4624–31. doi: 10.1016/j.vaccine.2013.07.040 (PMC3898716; doi:10.1016/j.vaccine.2013.07.040)
Supplement: Table S1 — Sequences of antigen 85A overlapping peptides. The table shows the sequences of 15mers overlapping by 10 amino acids, covering the sequence of Mtb antigen 85A. [file mmc1.docx]

**Supplementary Table 1. Sequences of antigen 85A overlapping peptides**

| 1. MQLVDRVRGAVTGMS | 34. VGLSMAASSALTLAI |
| --- | --- |
| 1. RVRGAVTGMSRRLVV | 35. AASSALTLAIYHPQQ |
| 1. VTGMSRRLVVGAVGA | 36. LTLAIYHPQQFVYAG |
| 1. RRLVVGAVGAALVSG | 37. YHPQQFVYAGAMSGL |
| 1. GAVGAALVSGLVGAV | 38. FVYAGAMSGLLDPSQ |
| 1. ALVSGLVGAVGGTAT | 39. AMSGLLDPSQAMGPT |
| 1. LVGAVGGTATAGAFS | 40. LDPSQAMGPTLIGLA |
| 1. GGTATAGAFSRPGLP | 41. AMGPTLIGLAMGDAG |
| 1. AGAFSRPGLPVEYLQ | 42. LIGLAMGDAGGYKAS |
| 1. RPGLPVEYLQVPSPS | 43. MGDAGGYKASDMWGP |
| 1. VEYLQVPSPSMGRDI | 44. GYKASDMWGPKEDPA |
| 1. VPSPSMGRDIKVQFQ | 45. DMWGPKEDPAWQRND |
| 1. MGRDIKVQFQSGGAN | 46. KEDPAWQRNDPLLNV |
| 1. KVQFQSGGANSPALY | 47. WQRNDPLLNVGKLIA |
| 1. SGGANSPALYLLDGL | 48. PLLNVGKLIANNTRV |
| 1. SPALYLLDGLRAQDD | 49. GKLIANNTRVWVYCG |
| 1. LLDGLRAQDDFSGWD | 50. NNTRVWVYCGNGKPS |
| 1. RAQDDFSGWDINTPA | 51. WVYCGNGKPSDLGGN |
| 1. FSGWDINTPAFEWYD | 52. NGKPSDLGGNNLPAK |
| 1. INTPAFEWYDQSGLS | 53. DLGGNNLPAKFLEGF |
| 1. FEWYDQSGLSVVMPV | 54. NLPAKFLEGFVRTSN |
| 1. QSGLSVVMPVGGQSS | 55. FLEGFVRTSNIKFQD |
| 1. VVMPVGGQSSFYSDW | 56. VRTSNIKFQDAYNAG |
| 1. GGQSSFYSDWYQPAC | 57. IKFQDAYNAGGGHNG |
| 1. FYSDWYQPACGKAGC | 58. AYNAGGGHNGVFDFP |
| 1. YQPACGKAGCQTYKW | 59. GGHNGVFDFPDSGTH |
| 1. GKAGCQTYKWETFLT | 60. VFDFPDSGTHSWEYW |
| 1. QTYKWETFLTSELPG | 61. DSGTHSWEYWGAQLN |
| 1. ETFLTSELPGWLQAN | 62. SWEYWGAQLNAMKPD |
| 1. SELPGWLQANRHVKP | 63. GAQLNAMKPDLQRAL |
| 1. WLQANRHVKPTGSAV | 64. AMKPDLQRALGATPN |
| 1. RHVKPTGSAVVGLSM | 65. LQRALGATPNTGPAP |
| 1. TGSAVVGLSMAASSA | 66. GATPNTGPAPQGA |
